# Optimization for a New XY Positioning Mechanism by Artificial Neural Network-Based Metaheuristic Algorithms

**DOI:** 10.1155/2022/9151146

**Published:** 2022-12-01

**Authors:** Minh Phung Dang, Hieu Giang Le, Ngoc Phat Nguyen, Ngoc Le Chau, Thanh-Phong Dao

**Affiliations:** ^1^Faculty of Mechanical Engineering, Ho Chi Minh City University of Technology and Education, Ho Chi Minh City, Vietnam; ^2^Faculty of Mechanical Engineering, Industrial University of Ho Chi Minh City, Ho Chi Minh City, Vietnam; ^3^Division of Computational Mechatronics, Institute for Computational Science, Ton Duc Thang University, Ho Chi Minh City, Vietnam; ^4^Faculty of Electrical & Electronics Engineering, Ton Duc Thang University, Ho Chi Minh City, Vietnam

## Abstract

This paper devotes a new method in modeling and optimizing to handle the optimization of the XY positioning mechanism. The fitness functions and constraints of the mechanism are formulated via proposing a combination of artificial neural network (ANN) and particle swarm optimization (PSO) methods. Next, the PSO is hybridized with the grey wolf optimization, namely PSO-GWO, which is applied to three scenarios in handling the single objective function. In order to search the multiple functions for the mechanism, the multiobjective optimization genetic algorithm (MOGA) is applied to the last scenario. The achieved results showed that the fitness functions are well-formulated using the PSO-based ANN method. In the scenario 1, the stroke achieved by the PSO-GWO (1852.9842 *μ*m) is better than that gained from the GWO (1802.8087 *μ*m). In the scenarios 2, the stress gained from the PSO-GWO (243.3183 MPa) is lower than that achieved from the GWO (245.0401 MPa). In the scenario 3, the safety factor retrieved from the PSO-GWO (1.9767) is greater than that achieved from the GWO (1.9278). In the scenario 4, by using MOGA, the optimal results found that the stroke is about (1741.3 *μ*m) and the safety factor is 1.8929. The prediction results are well-fitted with the numerical and experimental verifications. The results of this paper are expected to facilitate the synthesis and analysis of compliant mechanisms and related engineering designs.

## 1. Introduction

An advancement of micro/nanoscience and technology has a critically important significance in reducing the cost of products and increasing accurate working ability. In order to handle micro/nanoobjects, micro/nanomanipulators has become an essential demand. Micro/nanotechnologies have attended in various fields such as micromanipulation [1], micro-electromechanical system [2], micro/nanoindentation [3].

Especially in the field of micromanipulation, ultra-high positioners [4, 5] and grippers [6, 7] are primary applications. The positioners are often utilized to transfer a force from an actuator (e.g., piezoelectric actuator [8], electromagnetic actuator [9], electrostatic actuator [10]) toward a mechanical system. A sample is often mounted on the movable platform of the positioner, and the main assignment of the positioner is to locate the sample from an initial position to another one with micro/nanodisplacement steps [11]. Meanwhile, the grippers also perform a gripping task of a micro-object to a desirable location. A basic application of the positioners can be found in the indentation system, and the grippers are searched in DC assemble [12]. Moreover, a 3-DOF micromanipulation stage was developed to achieve a large rotational displacement [13]. In this study, the lever amplifier was utilized to enlarge the working stroke, and then the pseudo-rigid body method was applied to formulate the mathematical formulas of stiffness, displacement amplification ratio, and kinematics of the stage. Meanwhile, the dynamic performance of the stage was modeled through Lagrange's method. The precision and accuracy in transferring the motions of micromanipulations are directly affected by mechanical part systems. Basically, the rigid mechanical systems often use kinematic joints in connecting rigid links, as well as actuators and sensors in transforming the force/moment and sensing positions. Such rigid systems exist backlash and need more lubrications. So, the precision and accuracy of the positioner and grippers are decreased.

Unlike the rigid-based mechanical systems, the positioners based on compliant mechanisms and flexure hinges can achieve to a higher precision because compliant mechanism utilizes a single structure without backlash and friction. The operator of compliant mechanisms is replied on flexure hinges. The manufacturing of these mechanisms are benefits in cost and maintenance, since they can be fabricated by advanced techniques such as wire electrical discharged machining or additive manufacturing technology.

The most important aspect of developing compliant mechanism-based positioners is concentrated on modeling their behaviors (e.g., displacement/stroke, fatigue, parasitic motion, stress, and so forth). Until now, many analytical modeling methods have been proposed, e.g., pseudo-rigid-body model (PRBM) [14], dynamic stiffness matrix [15], and beam-constraint model [16]. More generally, according to the survey on the compliant mechanisms [17], many design synthesis and analysis methods were well-summarized, including PRBM, Castigliano's theory, compliance method, beam theory, and Ryu's method. These techniques are still helpful in the design synthesis of compliant mechanisms. However, a large deflection and complex configurations are mainly restricted problems in modeling procedures. More practically, the deformations of compliant mechanisms are often large. The analytical methods can analyze the large deflections, but they may be limited for analyzing a complex structure or irregular shape. In comparison with the analytical methods, finite element method (FEM) was proved as an efficient technique in analyzing a large deflection [18]. For solving a complex structure, i.e., parallel-connected series mechanisms, curvilinear structure, or irregular shape, FEM was an effective tool for compliant mechanism [19]. In modeling the nonlinear characteristics/large deformations of compliant mechanism-based positioner, a new intelligent modeling approach is proposed herein as an alternative for the analytical modeling techniques in this article.

In improving the characteristics of compliant mechanism-based positioners, two popular methods are usually used, including the experimental optimization method and metaheuristic optimization techniques. In the first type, traditional optimization methods, so-called nonmetaheuristic algorithms, gradient descent, Newton's method, and Taguchi method, response surface method, grey relational analysis, utility method [20, 21]. However, these nonmetaheuristics lead to a local optimum set. In the second type, the metaheuristic optimization methods, such as genetic algorithm (GA) [22, 23], particle swarm optimization (PSO) [24], cuckoo search algorithm [25], Dingo optimizer [26], and so on are required as an alternative choice to achieve a global optimum solution. More recently, a few efficient approaches suggested for optimizing in this field. A multiobjective genetic algorithm was proposed to optimize the flexure constant-force module [27]. The optimal parameters of a spatial constant-force end-effector were optimized through particle swarm optimization [28]. To improve the convergence speed and ability of searching the global optimum values, the PSO is coupled with the grey wolf optimization (GWO) [29]. The main target of the PSO-hybridized GWO optimizer is to get the capability of exploitation in the PSO and the capability of exploration in GWO in finding the global solutions. In recent years, many nature-inspired algorithms have suggested enhancing the capacity of optimizers in balancing the exploration and exploitation, such as Monarch butterfly optimization [30], Slime mould algorithm [31], Moth search algorithm [32], Hunger games search [33], Runge–Kutta method [34], colony predation algorithm [35], weighted mean of vectors [36], and Harris hawks optimization [37] Among these optimizers, the PSO-hybridized GWO is a relatively effective optimizer in solving the global optimum problems. Besides, the metaheuristic optimization algorithms have been used to improve the regression ability of artificial neural network (ANN), such as GA-ANN [38] and PSO-ANN [39].

In the literature, there are proposals in designing for compliant micro/nanopositioners, but a new design synthesis method is still essential. To overcome the limitations of the analytical methods and the local optimization methods, this paper proposes a new modeling and optimizing method in designing the compliant-based XY positioning mechanism. The main contribution of this study is to introduce the artificial neural network into the dimension synthesis of compliant mechanisms which is an assistant tool for further developing intelligent algorithms into the analysis and synthesis of compliant mechanisms.

First of all, a new structure of the mechanism is designed. And then, the fitness functions describing input design parameters and the output characteristics of the mechanism are modeled via using a combination of artificial neural network and particle swarm optimization. After that, the grey wolf optimization is hybridized with the particle swarm optimization to find some optimization scenarios for the mechanism. The results are also verified by simulations and experiments. The following organization of the paper are included.  Section 2 gives an introduction of mechanical structure and optimization problems for the mechanism.  Section 3 presents the research method.  Section 4 describes the achieved results. At last, Section 5 concludes the paper with the further study.

## 2. Structural Optimization Problem of XY Monolithic Mechanism

### 2.1. Mechanical Design

**Figure 1:**
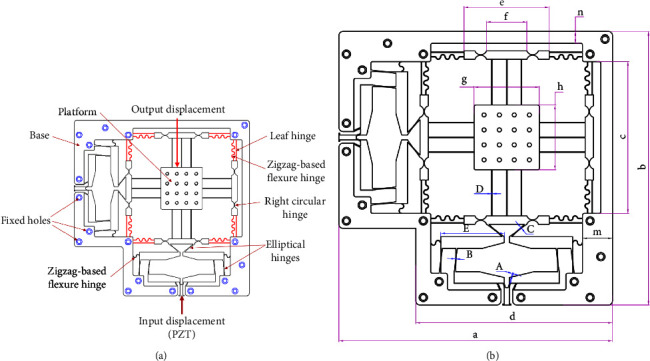
Mechanical scheme of proposed XY monolithic mechanism: (a) XY stage and (b) design parameters.

The new compliant XY stage was based on four-lever displacement amplifier integrated elliptical hinges and parallel guiding according to zigzag-based flexure springs and leaf hinges, as illustrated in Figure 1(a). Through many initial simulations, it is noted that the zigzag-based flexure springs offer the deflections better than that of leaf hinges. Additionally, the zigzag type is combined with leaf hinges so as to eliminate the parasitic motion error. Therefore, the zigzag type is chosen for designing the mechanism. In order to generate a large workspace for the XY stage, the lever amplifier is chosen because this amplifier has a simple structure and easy manufacture. In addition, two levers are arranged in symmetrical architecture so as to guarantee a translation motion in *x* axis or *y* axis with a larger stroke. In this study, the certain angle to the transverse and axial directions in the lever-type compliant amplifiers are aimed to reduce the mass of the amplifier, i.e., decreasing in unessential cross section of lever amplifier but still ensure a large amplification ratio. The XY stage is usefully potential for locating the specimens in a nanoindentation tester system. The material Al 7075 is selected for the proposed stage. This material possesses a few good properties such as a yield strength of 503 MPa, Young's modulus of 71700 MPa, density of 2810 kg/m^3^, and Poisson's ratio of 0.33. By checking initial input displacement through the FEM simulations, specification of XY stage is supposed that input displacement is 120 *μ*m. The parameters of XY stage is proposed in Figure 1(b). It consists of elements as follows: (i) nineteen holes are utilized to fix the stage on an unvibration table, (ii) a piezoelectric actuator (PZT) is combined with translational crew to generate the input displacement for the stage. The symmetric four-lever amplifier is integrated with elliptical hinges to generate a large stroke. The entire dimension of the stage is approximately 328 mm × 328 mm × 10 mm. In this research, elliptical hinges are proposed to integrate into XY stage because its benefits (e.g., minimal rotation axis shift, good safety factor, and large angular deflection) [40]. Besides, the zigzag-based flexure hinges and leaf hinges are developed and integrated in the stage in order to reduce parasitic motion and obtain more large displacement. The main geometric parameters of the proposed XY stage are provided in Table 1. Therefore, some main parameters of the stage should be optimized in improving the quality characteristics of the XY stage.

### 2.2. Formulation of Optimization Problems

Based on the proposed scheme of the XY monolithic mechanism (see in Figure 1), the initial simulations determine that there are five main design parameters affecting the entire performances of the mechanism. The first parameter is the thickness of hinge *A* being located at the first lever amplifier. The second parameter is the thickness of hinge *B* which is utilized to transfer the motions from the first lever amplifier to the second lever amplifier. The third parameter is the thickness of hinge *C* locating at the second lever amplifier. The fourth parameter is the length *E* of the second lever amplifier. The fifth parameter is the thickness of hinge *D* which is employed to transfer the motion from the zigzag driving mechanism to the middle shuttle platform. The ratio of length of the level is much sensitive to the static and dynamic performances of compliant mechanisms. If the length is increased, the entire size of the system is also increased. Hence, it is assumed that the length ignored the length of lever during the optimization because this work is aimed to design a compact size for the mechanism which is tended to be integrated into the in-situ nanoindentation tester.

It is observed that the proposed XY mechanism should possess multiple excellent performances, namely long fatigue life, large stroke, high safety factor, small stress, high resonant frequency, minimal parasitic motion, and so on. In the scope of this article, the three main performances are considered, including the large stroke, the high safety factor, and the minimal stress. For many different aspects in a few practical applications, the single-objective optimization concerning scenarios #1, 2, 3 are desired for the mechanism while the scenario #4 handles multi-performances for the mechanism, simultaneously. In this paper, the particle swarm optimization (PSO) [41] is combined with Grey Wolf Optimizer (GWO) [42] optimizer, so-called PSO-GWO [43], is employed to solve the single objective optimization problems for scenario 1–3. Three numerical problems are considered to demonstrate the efficiency of the proposed optimizer as follows.

#### 2.2.1. Scenario #1

Find design vector: **x**=[*x*_1_, *x*_2_, *x*_3_, *x*_4_, *x*_5_]

(1) $$\begin{array}{c} \text{Maximize}:F_{1}\left(x\right)\text{.} \end{array}$$

Range of design variables (unit: mm):

(2) $$\begin{array}{c} \left\{\begin{array}{c} 0.9\leq x_{1}\leq 1.1\text{,}\\ 0.5\leq x_{2}\leq 0.6\text{,}\\ 0.5\leq x_{3}\leq 1.0\text{,}\\ 0.5\leq x_{4}\leq 0.6\text{,}\\ 73\leq x_{5}\leq 78\text{.} \end{array}\right. \end{array}$$

#### 2.2.2. Scenario #2

Find design vector: **x**=[*x*_1_, *x*_2_, *x*_3_, *x*_4_, *x*_5_]

(3) $$\begin{array}{c} \text{Minimize}:F_{2}\left(x\right)\text{.} \end{array}$$

Range of design variables (unit: mm):

(4) $$\begin{array}{c} \left\{\begin{array}{c} 0.9\leq x_{1}\leq 1.1\text{,}\\ 0.5\leq x_{2}\leq 0.6\text{,}\\ 0.5\leq x_{3}\leq 1.0\text{,}\\ 0.5\leq x_{4}\leq 0.6\text{,}\\ 73\leq x_{5}\leq 78\text{.} \end{array}\right. \end{array}$$

#### 2.2.3. Scenario #3

Find design vector: **x**=[*x*_1_, *x*_2_, *x*_3_, *x*_4_, *x*_5_]

(5) $$\begin{array}{c} \text{Maximize}:F_{3}\left(x\right)\text{.} \end{array}$$

Range of design variables (unit: mm):

(6) $$\begin{array}{c} \left\{\begin{array}{c} 0.9\leq x_{1}\leq 1.1\text{,}\\ 0.5\leq x_{2}\leq 0.6\text{,}\\ 0.5\leq x_{3}\leq 1.0\text{,}\\ 0.5\leq x_{4}\leq 0.6\text{,}\\ 73\leq x_{5}\leq 78\text{,} \end{array}\right. \end{array}$$

where *x*_*1*_, *x*_*2*_, *x*_*3*_, *x*_*4*_, and *x*_*5*_ are the design parameters corresponding to *A, B, C, D*, and *E*. The considered objective functions consist of the stroke *F*_*1*_(**x**), the stress *F*_*2*_(**x**), and the safety factor *F*_*3*_(**x**).

In a real product, multiple functions of the mechanism are simultaneously required. Besides, the stroke is often conflicted with the safety factor or the stress. Therefore, the multiobjective optimization genetic algorithm (MOGA) optimizer [44] is utilized to solve a tradeoff between the stroke and the safety factor. The multiple-objective optimization problem for the mechanism is defined by scenario #4.

#### 2.2.4. Scenario #4

Find design vector: **x**=[*x*_1_, *x*_2_, *x*_3_, *x*_4_, *x*_5_]

(7) $$\begin{array}{c} \left\{\begin{array}{c} \text{Maximize}:F_{1}\left(x\right)\text{,}\\ \text{Maximize}:F_{3}\left(x\right)\text{.} \end{array}\right. \end{array}$$

Subject to constraints:

(8) $$\begin{array}{c} F_{3}\left(x\right)\geq 1.8\text{.} \end{array}$$

Range of design variables (unit: mm):

(9) $$\begin{array}{c} \left\{\begin{array}{c} 0.9\leq x_{1}\leq 1.1\text{,}\\ 0.5\leq x_{2}\leq 0.6\text{,}\\ 0.5\leq x_{3}\leq 1.0\text{,}\\ 0.5\leq x_{4}\leq 0.6\text{,}\\ 73\leq x_{5}\leq 78\text{.} \end{array}\right. \end{array}$$

## 3. Research Method

**Figure 2:**
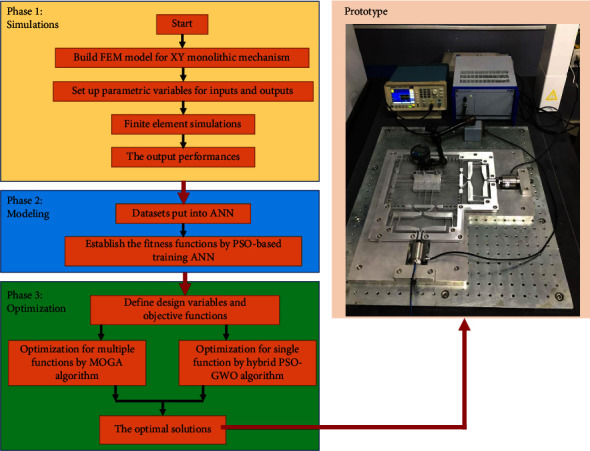
Flowchart of proposed ANN-based metaheuristic algorithms for modeling and optimization.

This part provides a new approach in optimizing the performances of the XY positioning mechanism. In this context, the proposed method is developed with three subphases. In the first subphase, the simulations for the mechanism are carried out by constructing a finite element model, setup parametric variables for inputs and outputs, perform finite element simulations, and collect datasets. In the second subphase, the data are put input into the ANN program, and the particle swarm optimization (PSO) algorithm is employed to train the artificial neural network (ANN). In the last subphase, the output performances are optimized by the PSO-hybridized with grey wolf optimization (GWO) algorithm. The flowchart of modeling and optimization for the mechanism is given in Figure 2. Details of three subphases are presented below.

The present study is aimed to recommend the ANN-based PSO into the dimension synthesis of compliant mechanisms. The presented method can be the efficient assistance for further developing intelligent algorithms into the analysis and synthesis of engineering design.

### 3.1. Simulation of XY Monolithic Mechanism

**Figure 3:**
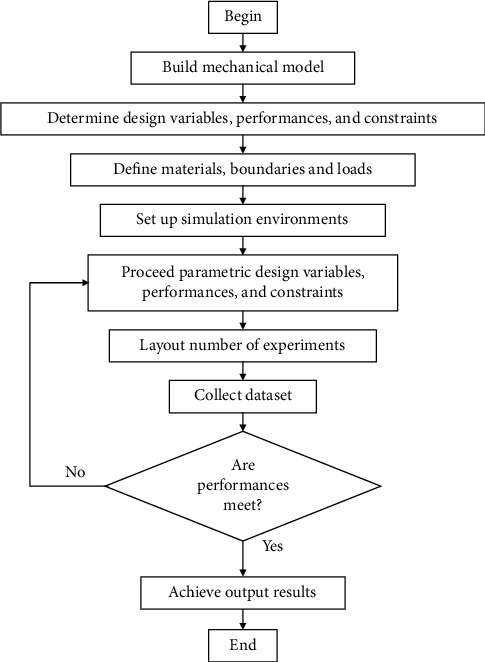
Simulation scheme of XY monolithic mechanism.

In order to collect the performances of the mechanism, this section simulates the mechanism through the following detailed multiple-steps, as shown in Figure 3.

- The mechanical model of the XY positioning mechanism is initialized.
- The key design variables (*A, B, C, D, E*), output performances and constraints (stroke, safety factor, and stress), are determined.
- Regarding material, Al-7075 T73 is employed.
- The boundaries conditions are setup, and a load of N from the piezoelectric actuator is applied to the input port of the mechanism (see Figure 1).
- The simulation environments are setup regarding the nonlinear characteristics in simulations. The nonlinearity means a large deformation of hinges.
- The variables (inputs, outputs, and constraints) are parametric.
- The 27 numerical experiments are planned, and the datasets are collected.
- If the performances are satisfied with the initial requirements, the process is ended herein. Otherwise, it goes back to proceed the parametric variables.

### 3.2. Artificial Neural Network Algorithm

The performances of the mechanism are modeled using the artificial neural network (ANN) method [45, 46]. The ANN is often utilized as modeling technique based on the reasoning of human brain. The ANN is considered as a basic type of deep learning method. A basic ANN includes neuron nodes, layers (input, hidden, and output), weights, bias, and activation functions. A primary formula of ANN is formulated as follows.

(10) $$\begin{array}{c} z=f\left(b+\overset{N}{\underset{i=1}{\sum }}w_{i}x_{i}\right)\text{,} \end{array}$$

where *z* is the output value of *k*^th^ neuron node, *b* is bias, *W* is weight, and *x* is input node. *N* is number of input nodes.

In this work, there are five main inputs, the design parameters of the mechanism (*A, B, C, D, E*), the number of nodes (*n*_node_) in a hidden layer is computed by following formula.

(11) $$\begin{array}{c} n_{no\;de}=inputs*2+outputs\text{.} \end{array}$$

**Figure 4:**
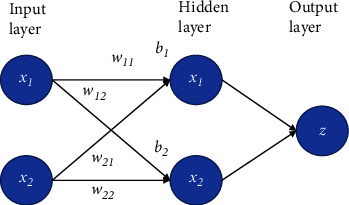
Primary ANN architecture.

In this study, the feedforward algorithm is employed for training the ANN architecture. An ANN architecture is primarily illustrated as in Figure 4.

### 3.3. Modeling the Performances Using the PSO-ANN Algorithm

The effectiveness and accuracy of an ANN architecture depends on several components such as training algorithm, activation function, number of neurons, number of hidden layers, weight, and bias. In the limitation of this context, the bias and weight of the feedforward ANN algorithm are optimized by the PSO optimizer [47]. The mean squared error (MSE) is considered as the objective function of the PSO algorithm, which is described by the formula as.

(12) $$\begin{array}{c} \text{MSE}=\frac{1}{m}{\overset{m}{\underset{i=1}{\sum }}\left(p_{i}-{\hat{p}}_{i}\right)}^{2}\text{,} \end{array}$$

where *p* is the simulated value and $\hat{p}$ is the estimated value, and *m* is input size.

The PSO optimizer is aimed to update the weights and bias to determine the best ANN architecture. This method goes several steps in pseudocode of Algorithm 1.

### 3.4. Optimization for XY Positioning Mechanism by PSO-Hybridized GWO

**Figure 6:**
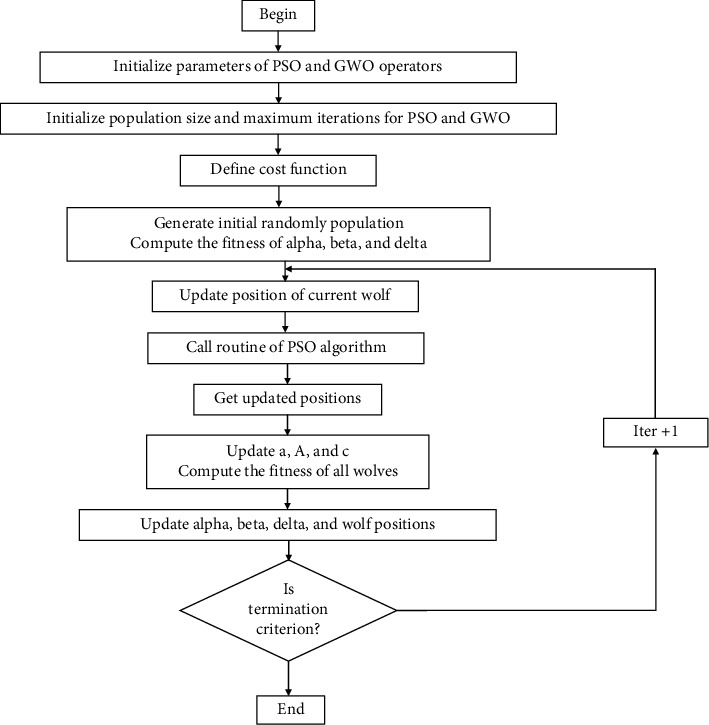
Basic procedure of PSO-hybridized GWO.

**Figure 7:**
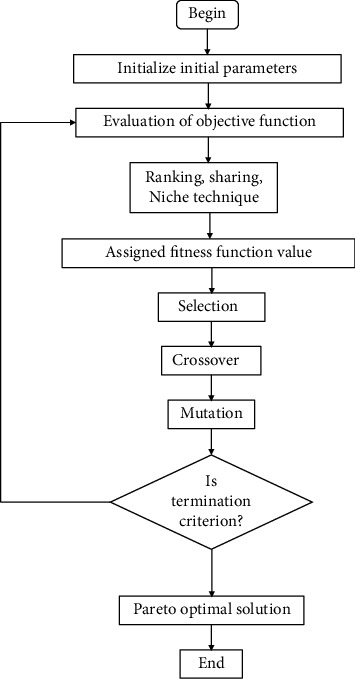
Operating flowchart of multiobjective optimization algorithm.

In order to solve the four scenarios of the XY positioning mechanism (see in section 2), the three first scenarios in equations (1)–(3) are handled by the PSO-hybridized GWO algorithm [43], as depicted in Figure 6. The last scenario in (4) is solved by the MOGA optimizer [44], as demonstrated in Figure 7.

The pseudocodes for the PSO-GWO algorithm and the MOGA are shown in Algorithm 2 and Algorithm 3, respectively.

## 4. Results and Discussion

### 4.1. Simulation Results of XY Positioning Mechanism

In this part, the numerical simulations are conducted to achieve the datasets which are employed for the modeling and optimizing of the mechanism. In this article, there are five main parameters (*A, B, C, D, E*) directly affecting the stroke, stress, and safety factor of the proposed mechanism. Based on the number of design variables, the twenty-seven numerical design points are planned. An input displacement of 120 *μ*m from the actuator is applied to the input part, the output performances (stroke, stress, and safety factor) are collected. Considering the finite element simulations, a nonlinear analysis is employed. The boundary conditions and loads are recalled in Figure 1. Ten-node tetrahedral elements are employed for the mesh. The results found that there are about 149242 elements and 275268 nodes. The mesh metrics is measured through Skewness criterion to ensure the analysis convergence. The entire inputs and outputs are resolved as parametric variables. The datasets are given in Table 2.

The results of Table 2 remarked that the safety factor is increased when the stress is correspondingly decreased. It is also noted that the stroke is always conflicted with the stress. The stress is considered as equivalent von Mises stress.

### 4.2. Modeling Results by PSO-Based ANN

First of all, the data in Table 1 are embedded into the feedforward ANN program. The data are separated into the training, testing, and validating subsets. In this article, a hidden layer is suggested for the ANN architecture. By using the (6), the number of nodes of hidden layers is equal to 11. Subsequently, the formulated ANN program is transferred to the PSO optimizer to make a hybrid PSO-ANN algorithm. The main parameters of the PSO operator consist of population size of 50, inertia weight of 1, inertia weight damping ratio of 0.9, personal learning coefficient of 1.5, global learning coefficient of 2, and max iteration of 200. The PSO is applied to minimize the MSE value of the ANN and determine the optimal values of weights and bias. Summarily, the hybrid PSO-ANN algorithm is extended to build the fitness functions and constraints for the XY positioning mechanism. The proposed PSO-ANN code is performed in MATLAB R2019b.

**Figure 8:**
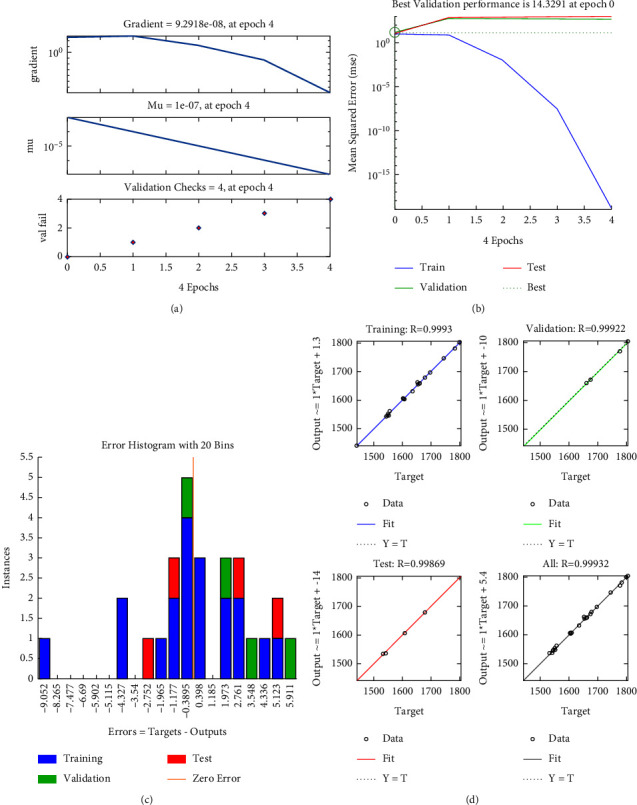
PSO-ANN for stroke: (a) training, (b) performance, (c) histogram, and (d) correlation coefficient.

Figure 8 presents the modeling results for the stroke. It includes the training state, performance, error histogram, and training process in Figures 7(d), respectively. The results found that the correlation coefficients (*R*) of the stroke's ANN modeling for the entire data, training, testing, and validating are approximately 1. This means that the built ANN architecture is a reliable and accurate tool in modeling the stroke.

**Figure 9:**
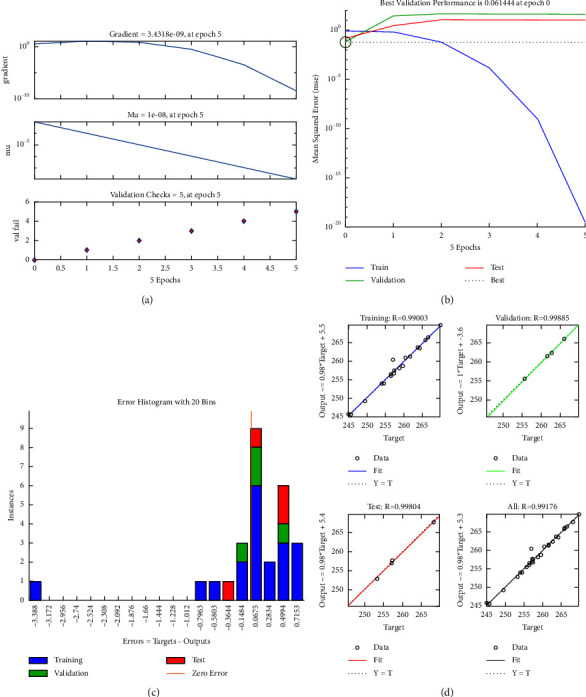
PSO-ANN for stress: (a) training, (b) performance, (c) histogram, and (d) correlation coefficient.

The second step is focused on developing the ANN structure for the stress. The PSO-ANN hybridization is also applied to model the stress. The training, performance, error histogram, and correlation coefficient are provided in Figures 9(a)–9(d). It showed that the ANN structure has a high efficiency in modeling the stress with the correlation coefficients being close to 1.

**Figure 10:**
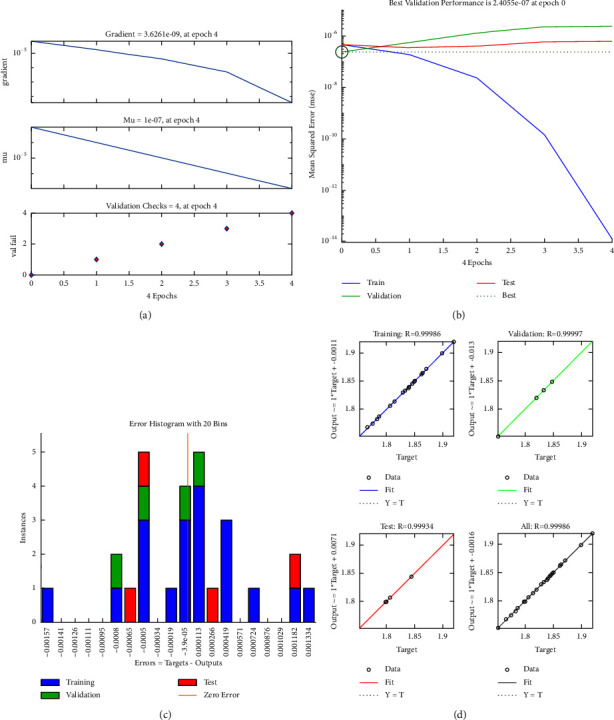
PSO-ANN for safety factor: (a) training, (b) performance, (c) histogram, and (d) correlation coefficient.

Lastly, the safety factor is modeled via using the PSO-ANN algorithm. The results indicated that the modeling of safety factor has a high accuracy with good correlation coefficients of entire data, training, testing, and validating being nearly 1, as depicted in Figure 10.

Besides, the mean squared error (MSE), root mean squared error (RMSE), and coefficient of determination (*R*^2^) for each model are calculated and given in Table 3. The results note that the *R*^2^ values are almost close to one. To sum up, this section establishes the fitness function and constraints with a high accuracy for the XY positioning mechanism.

### 4.3. Optimization Results

This part presents the optimization process and the optimal solutions. The proposed PSO-GWO hybridization is carried out to solve the single objective function in three first scenarios (1–3) in MATLAB R2019b. The key parameters of this optimizer include an initial population of 30, *c*_1_ of 0.5, *c*_2_ of 0.5, and *c*_3_ of 0.5. The whole of the fitness functions from the established PSO-ANN are embedded into the PSO-GWO. Regarding the optimization of the scenario 1 in equation (1), a max iteration of 200 is set up for optimizing the stroke. For the scenario 2 in equation (2), a maximum iteration of 500 is set for minimizing the stress. For the scenario 3 in equation (3), a max iteration of 1500 is set for maximizing the safety factor. The results are summarized in Table 4.

Considering the scenario 1 (i.e., maximize the stroke), the stroke achieved from the PSO-GWO hybridization (1852.9842 *μ*m) is a little better than the one gained from the GWO (1802.8087 *μ*m). Regarding the scenarios 2 (i.e., minimize the stress), the stress got from the PSO-GWO hybridization (243.3183 MPa) is smaller than that got from the GWO (245.0401 MPa). Considering the scenario 3 (i.e., maximize the safety factor), the safety factor gained from the PSO-GWO (1.9767) hybridization is greater than that achieved from the GWO (1.9278). Besides, it found that the PSO-GWO improved optimization strategy is superior to the original GWO in terms of computational efficiency (i.e., fast convergence speed) and better results.

In order to solve the scenario 4 (i.e., maximize the stroke and maximize the safety factor simultaneously), the MOGA optimizer is applied. The optimal results found that the stroke is about (1741.3 *μ*m) and the safety factor is equal to 1.8929. Finally, the optimal design variables are found *A* = 0.9 mm, *B* = 0.5 mm, *C* = 0.5 mm, *D* = 0.5 mm, *E* = 73 mm, as given in Table 4.

**Figure 11:**
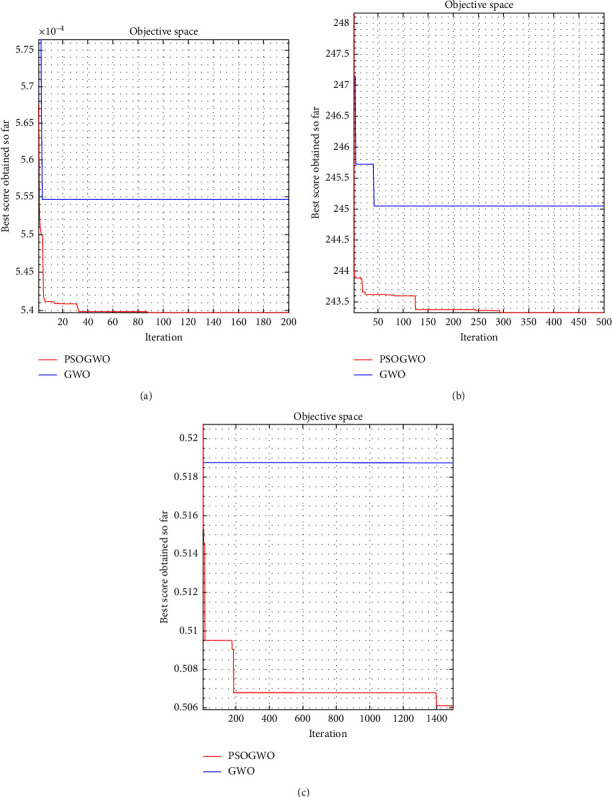
Convergent plots: (a) stroke, (b) stress, and (c) safety factor.

The convergent histories of the scenario 1 (stroke), the scenario 2 (stress), and the scenario 3 (safety factor) achieved from the PSO-GWO hybridization and GWO are provided in Figure 11 (1-c), respectively.

To sum up, the optimal results in the four scenarios are totally satisfied with the initial design targets of the XY positioning mechanism.

### 4.4. Verification Results

First of all, the optimal results are verified by using finite element analysis in ANYS 2019R2 software. The comparison results showed that the optimal results achieved from the proposed optimizers are well-agreed with the verification results. In comparison with the initial design, the optimal performances of the XY positioning mechanism are largely improved when the MOGA optimizer is applied, as provided in Table 5.

Compared with the previous designs of XY mechanisms, the present design proposed a larger stroke than that of previous studies [48–50]. It is found that the entire dimension of the presented XY mechanism is bigger than that of the other designs, as given in Table 6. However, the presented XY mechanism has a large enough dimension for locating the testing material samples in nanoindentation devices.

**Figure 12:**
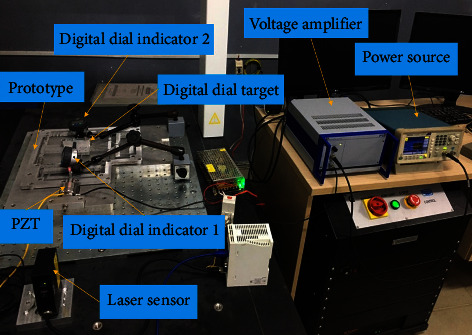
Experiments of the XY mechanism.

Finally, by using the optimal parameters (*A* = 0.9 mm, *B* = 0.5 mm, *C* = 0.5 mm, *D* = 0.5 mm, *E* = 73 mm), the prototype of the XY mechanism is fabricated by wire electrical discharged machining. The experiments are conducted to measure the stroke of the mechanism, as provided in Figure 12. The experimental results found that the stroke is about 1632 *μ*m. The error between the experimental result and optimal result is 6.27%. Therefore, this result is close to the predicted stroke from the MOGA (1741.3 *μ*m).

## 5. Conclusions

This paper has proposed a new modeling and optimizing approach applied to solve the optimization of the XY positioning mechanism. The fitness functions and constraints of the mechanism are built via proposing a combination of ANN and PSO method. The PSO helps to improve the accuracy of the ANN by finding the optimal weights and bias. Next, the PSO is hybridized with the GWO, namely PSO-GWO, is employed to three scenarios in solving the single objective function. In order to search the multiple functions for the mechanism, the MOGA method is applied to the scenario 4. The achieved results from the PSO-ANN, PSO-GWO, and MOGA methods are drawn as follows:

- The fitness functions are well-built through the PSO-based ANN method with the good metrics (MSE, RMSE, and *R*^2^).
- In the scenario 1, the stroke achieved by the PSO-GWO hybridization (1852.9842 (*μ*m)) is a little better than the one gained from the GWO (1802.8087 (*μ*m)).
- In the scenarios 2, the stress gained from the PSO-GWO hybridization (243.3183 (MPa)) is smaller than that achieved from the GWO (245.0401 (MPa)).
- In the scenario 3, the safety factor got from the PSO-GWO (1.9767) hybridization is greater than that achieved from the GWO (1.9278).
- In the scenario 4, the MOGA optimizer is applied. The optimal results found that the stroke is about (1741.3 *μ*m) and the safety factor is equal to 1.8929.
- The prediction by the proposed methods are relatively fit with the numerical and experimental verifications.

From the modeling and optimizing results, the proposed techniques proved a good ability to handle the design problems for different compliant mechanisms and other engineering problems in the future studies.

## Figures and Tables

**Figure 5:**
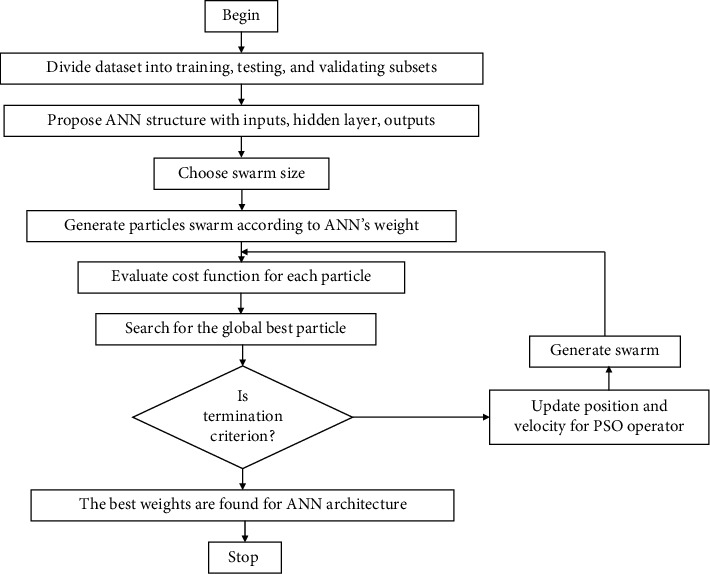
Basic scheme of PSO-based ANN training.

**Algorithm 1:**
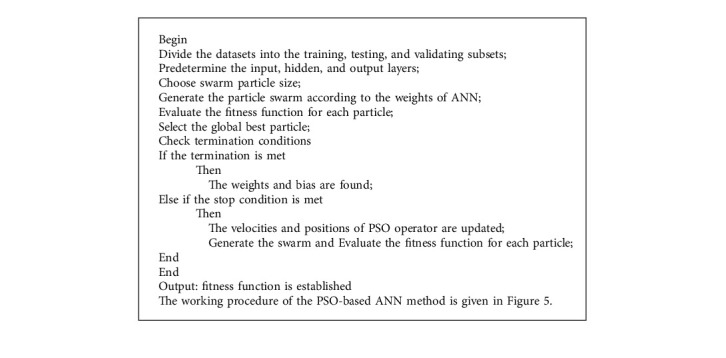
Pseudocode of PSO-based ANN.

**Algorithm 2:**
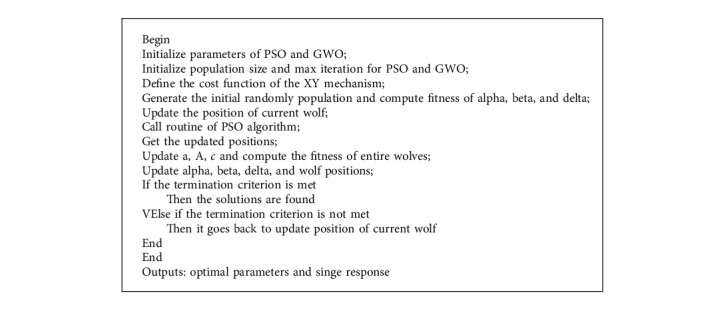
Pseudocode for the PSO-GWO.

**Algorithm 3:**
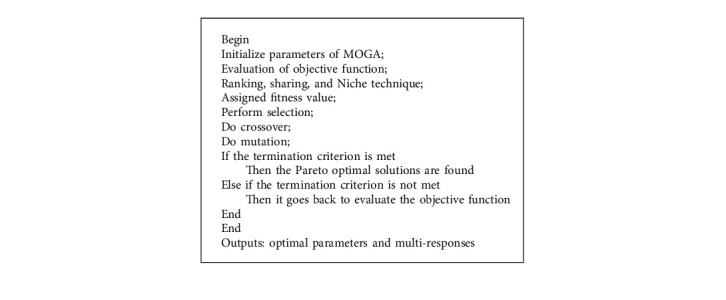
Pseudocode for the MOGA.

**Table 1:** The main geometrical dimensions of the XY stage (unit: mm).

Par.	Value	Unit
*a*	328	mm
*b*	328	mm
*c*	182	mm
*d*	236	mm
*e*	102	mm
*f*	48	mm
*G*	78	mm
*h*	78	mm
*m*	36	mm
*n*	14	mm
*A*	*0.9* *≤* *A* *≤* *1.1*	mm
*B*	*0.5* *≤* *B* *≤* *0.6*	mm
*C*	*0.5* *≤* *C* *≤* *1*	mm
*D*	*0.5* *≤* *D* *≤* *0.6*	mm
*E*	*73* *≤* *E* *≤* *79*	mm

**Table 2:** Simulation results for the XY positioning mechanism.

No.	*A* (mm)	*B* (mm)	*C* (mm)	*D* (mm)	*E* (mm)	Safety factor	Stress (MPa)	Stroke (*μ*m)
1	1	0.55	0.75	0.55	75.5	1.848	256.972	1635.051
2	0.9	0.55	0.75	0.55	75.5	1.843	253.278	1609.341
3	1.1	0.55	0.75	0.55	75.5	1.838	257.124	1655.087
4	1	0.5	0.75	0.55	75.5	1.839	257.340	1661.730
5	1	0.6	0.75	0.55	75.5	1.786	265.783	1606.541
6	1	0.55	0.5	0.55	75.5	1.861	253.969	1744.919
7	1	0.55	1	0.55	75.5	1.799	268.093	1552.369
8	1	0.55	0.75	0.5	75.5	1.833	259.613	1661.656
9	1	0.55	0.75	0.6	75.5	1.813	264.248	1603.002
10	1	0.55	0.75	0.55	73	1.828	261.586	1604.460
11	1	0.55	0.75	0.55	78	1.783	266.582	1653.056
12	0.9	0.5	0.5	0.5	78	1.850	256.438	1805.540
13	1.1	0.5	0.5	0.5	73	1.920	245.642	1783.775
14	0.9	0.6	0.5	0.5	73	1.870	249.432	1697.036
15	1.1	0.6	0.5	0.5	78	1.8063	258.736	1801.304
16	0.9	0.5	1	0.5	73	1.849	257.256	1550.759
17	1.1	0.5	1	0.5	78	1.846	255.675	1659.528
18	0.9	0.6	1	0.5	78	1.752	265.944	1547.772
19	1.1	0.6	1	0.5	73	1.833	262.755	1532.113
20	0.9	0.5	0.5	0.6	73	1.863	254.574	1679.535
21	1.1	0.5	0.5	0.6	78	1.798	260.385	1777.515
22	0.9	0.6	0.5	0.6	78	1.820	256.464	1675.118
23	1.1	0.6	0.5	0.6	73	1.899	244.976	1678.494
24	0.9	0.5	1	0.6	78	1.813	257.366	1555.992
25	1.1	0.5	1	0.6	73	1.805	261.642	1544.069
26	0.9	0.6	1	0.6	73	1.775	263.633	1441.013
27	1.1	0.6	1	0.6	78	1.766	269.777	1541.295

**Table 3:** The PSO-ANN modeling results for the mechanism.

PSO-based ANN models	Performance metrics			
	Datasets	MSE	RMSE	*R * ^2^
Stroke	Model	11.3014	3.36176	0.9986
	Training subset	10.8031	3.28681	0.9986
	Testing subset	10.6411	3.26207	0.9969
	Validating subset	14.3291	3.78538	0.9963

Stress	Model	0.6165	0.7852	0.9836
	Training subset	0.8323	0.9123	0.9801
	Testing subset	0.1463	0.3825	0.9952
	Validating subset	0.0614	0.2478	0.9801

Safety factor	Model	4.2363e-07	0.0007	0.9997
	Training subset	4.5127e-07	0.0007	0.9997
	Testing subset	4.7540e-07	0.0007	0.9986
	Validating subset	2.4055e-07	0.0005	0.9998

**Table 4:** The optimal results for four scenarios.

	Numerical examples	Optimal solutions (mm)	Stroke (*μ*m)	Stress (MPa)	Safety factor
GWO for single objective function	Scenario 1	*A* = 1.1, *B* = 0.6,*C* = 0.5, *D* = 0.5, *E* = 78	1802.8087	258.1356	1.8061
	Scenario 2	*A* = 1.1, *B* = 0.5,*C* = 0.5, *D* = 0.5, *E* = 78	1763.2	245.0401	1.8866
	Scenario 3	*A* = 1.056, *B* = 0.572, *C* = 0.559, *D* = 0.580, *E* = 75.878	1688.4	255.1731	1.9278

PSO-hybridized GWO for single objective function	Scenario 1	*A* = 0.953, *B* = 0.556, *C* = 0.5, *D* = 0.5, *E* = 78	1852.9842	261.3997	1.8235
	Scenario 2	*A* = 1.1, *B* = 0.537,*C* = 0.678, *D* = 0.5,*E* = 74.890	1672.6	243.3183	1.8815
	Scenario 3	*A* = 0.9, *B* = 0.571,*C* = 0.5, *D* = 0.546,*E* = 75.325	1790.9	256.0850	1.9767

MOGA for multiple-objective functions	Scenario 4	*A* = 0.9, *B* = 0.5, *C* = 0.5,*D* = 0.5, *E* = 73	1741.3	250.55	1.8929

**Table 5:** Comparison of the initial design with the optimal results.

Design method	Stroke (*μ*m)	Stress (MPa)	Safety factor
Initial design	1574.1	267.17	1.7734
Optimal solutions by MOGA	1741.3	250.55	1.8929

**Table 6:** Comparison of the present design with the previous designs.

Design method	Dimension	Stroke (*μ*m)	Stress (MPa)	Safety factor
Huang and Dao [48]	130.9 mm × 130.9 mm × 8 mm	115.16 *μ*m × 115.16 *μ*m	132.34	3.86
Zhao et al. [49]	156.2 mm × 156.2 mm	25 *μ*m × 25 *μ*m	37.7	10.98
Wang and Xu [50]	NA	700 µm × 700 *µ*m	496.7	1.01
Present design	328 mm × 328 mm × 10 mm	1741.3 *μ*m × 1741.3 *μ*m	250.55	1.8929

## Data Availability

The data used to support the findings of this study are included within the article.
